# Diverse Family Caregivers, Diverse Experiences in Caring

**DOI:** 10.1093/geroni/igaa057.083

**Published:** 2020-12-16

**Authors:** Heather Young, Janice Bell, Rita Choula, Tina Kilaberia, Susan Reinhard

**Affiliations:** 1 University of California Davis, Sacramento, California, United States; 2 AARP, Washington, District of Columbia, United States; 3 UC Davis Health, Sacramento, California, United States

## Abstract

The Home Alone Revisited Study examined the experience of family caregivers including demands of caring for someone with physical, mental or behavioral health care needs, revealing a large proportion of caregivers managing medical/nursing tasks. The aim of this paper was to explicate racial/ethnic differences in the prevalence of tasks and the perceptions of caregivers regarding their responsibilities. The study was a nationally representative, population-based online survey that oversampled underrepresented groups. The sample included 2,089 diverse family caregivers (Hispanic, n=307; Black/African American, n = 231; Asian/Pacific Islander, n=196; White, n=1,284; multi-racial, n= 71), 57% women, ranging from 18 to 91 years of age. Overall, fifty-two percent of caregivers reported performing at least one medical/nursing task for their care recipient, with a greater percentage (p<0.01) engaged in these tasks in two groups: Asian/Pacific Islander (62%) and multiracial (62%). Across the five racial/ethnic groups, we found significant differences in having a choice, feeling pressure, and the influence of culture and religion on the decision to take on medical/nursing tasks. There were also significant differences in the prevalence of performing 17 specific tasks, reports of difficulty with the tasks, the extent to which respondents felt prepared, and the strain that they experienced in providing care. The findings reveal both commonalities and significant heterogeneity in the caregiving experience, suggesting the importance of advancing the dialogue among health and social service providers about providing culturally and linguistically appropriate care and developing responsive tools and resources to support family caregivers as they engage in complex care.

